# Targeting macrophage-mediated TGF-β/BMP signaling in ankylosing spondylitis: from inflammation to pathological bone formation

**DOI:** 10.3389/fimmu.2026.1788883

**Published:** 2026-07-23

**Authors:** Shalayiding Aierxiding, Zheng Ren

**Affiliations:** 1 Department of Orthopedics, Xinjiang 474 hospital, Ürümqi, Xinjiang, China; 2 Department of Spine Surgery I, The Sixth Affiliated Hospital of Xinjiang Medical University, Ürümqi, Xinjiang, China; 3 Xinjiang Institute of Spinal Surgery, The Sixth Affiliated Hospital of Xinjiang Medical University, Ürümqi, Xinjiang, China

**Keywords:** ankylosing spondylitis, BMP, heterotopic ossification, macrophage polarization, osteoimmunology, spinal fusion, TGF-β

## Abstract

Ankylosing spondylitis (AS) is a chronic, immune-mediated disease characterized by inflammatory arthritis and pathological new bone formation, ultimately leading to spinal fusion and functional impairment. Despite effective suppression of inflammation by biologic agents targeting cytokines such as TNF-α and IL-17A, radiographic progression often continues, highlighting a critical dissociation between inflammatory activity and structural damage. The literature suggests that macrophage polarization and the TGF-β/BMP signaling axis collectively constitute a critical nexus linking inflammation to aberrant osteogenesis in AS. Moreover, it details how the unique entheseal microenvironment—shaped by biomechanical stress, hypoxia, and a distinct cytokine milieu—drives macrophages toward a spectrum of pro-osteogenic phenotypes through a mechano-inflammatory feedback loop. After that, polarized macrophages, in turn, serve as pivotal cellular engineers that locally activate and sustain TGF-β/BMP signals through proteolytic cleavage and acidic remodeling of the ECM. This self-amplifying loop directly orchestrates endochondral ossification at ligamentous insertion sites, culminating in syndesmophyte formation and spinal ankylosis. Therapeutically, this mechanistic understanding highlights promising avenues for disease modification beyond conventional anti-inflammatory strategies. Targeting macrophage polarization states or disrupting the TGF-β/BMP activation cascade may offer dual benefits—suppressing both inflammation and structural progression—and pave the way for genuine disease-modifying therapies in AS.

## 1 Introduction

Ankylosing spondylitis (AS) is a chronic inflammatory condition that predominantly affects the axial skeleton and is a quintessential example of axial spondyloarthritis (axSpA) (1). Chronic inflammatory back pain and structural changes in the sacroiliac joints are hallmarks of AS (2). Global epidemiological observations estimate that the prevalence of AS ranges from 0.1% to 1.4% (3). It typically manifests in early adulthood, particularly between ages 20 and 40, is more frequent in males, and is genetically associated with the human leukocyte antigen (HLA) B27 allele (4, 5). Nonetheless, a significant proportion of patients with AS lack this specific genetic marker, suggesting that additional genetic and non-genetic factors likely contribute to the disease’s pathogenesis (6). Pathologically, the disease is characterized by abnormal bone formation driven by inflammation and osteogenesis, particularly by cytokines such as tumor necrosis factor-α (TNF-α) and interleukin-17A (IL-17A) (7). Clinically, AS presents with chronic back pain, stiffness, and extra-articular manifestations such as uveitis and psoriasis (8, 9). Diagnosis is facilitated through clinical evaluation, radiographic imaging, and laboratory tests (10). Given the limitations associated with non-steroidal anti-inflammatory drugs (NSAIDs) in management strategies, current efforts are increasingly directed towards the development of targeted therapies that offer non-curative alternatives (11). Therapeutic options have evolved with the introduction of biologic medicines, notably TNF and IL-17A inhibitors; however, many patients continue to experience ongoing structural progression, underscoring the need for therapies targeting osteo-proliferative pathways (12). The current literature review reveals that continued research into disease mechanisms and innovative treatments is essential, especially for the effective management of AS by targeting abnormal bone formation.

**Figure 1:**
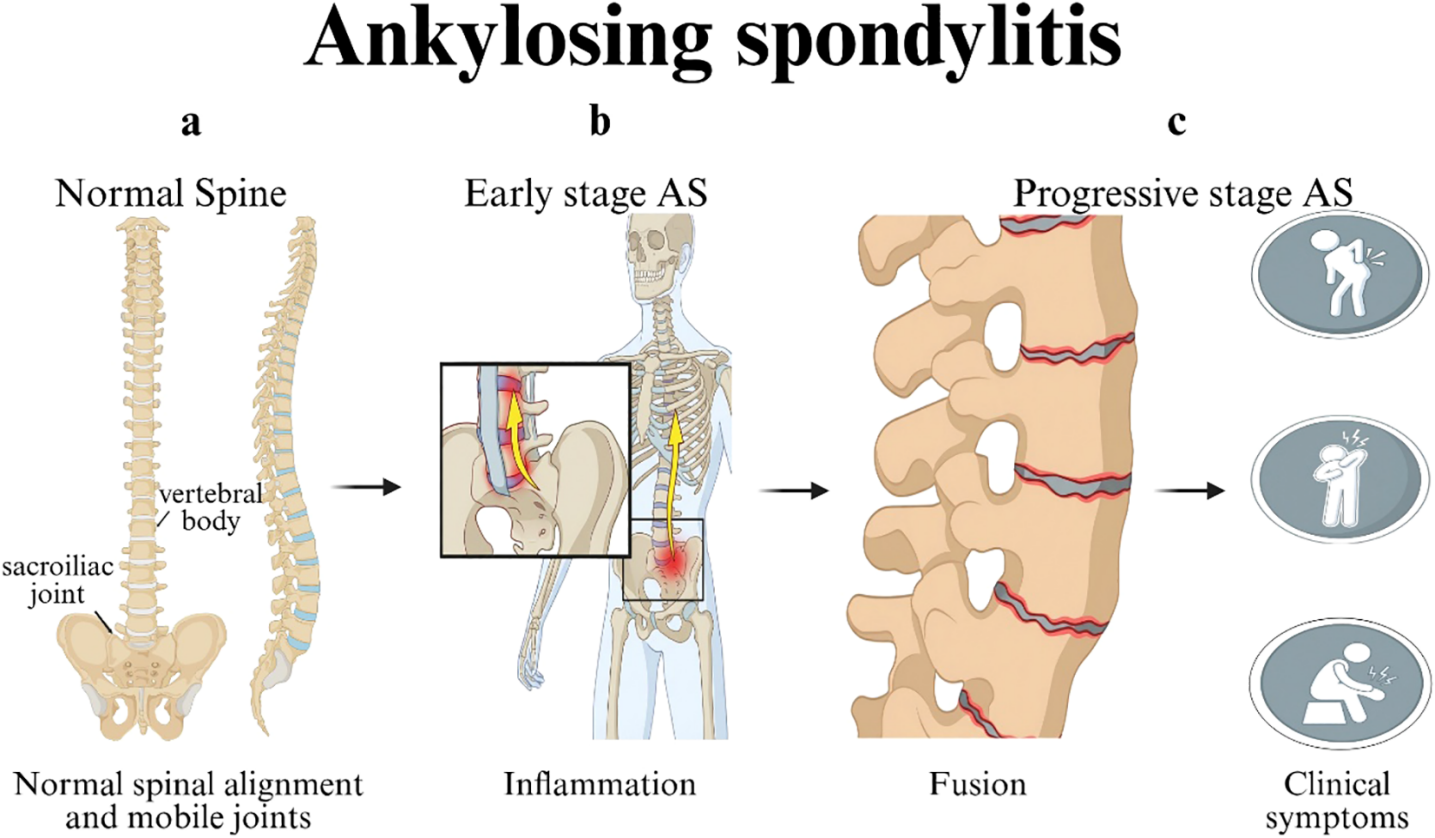
Progression of ankylosing spondylitis (AS) from normal spine to structural fusion. **(a)** Normal spine and mobile sacroiliac joints. **(b)** Early-stage inflammation with immune infiltration and enthesitis. **(c)** Advanced disease characterized by syndesmophyte formation, sacroiliac joint fusion, and spinal ankylosis (“bamboo spine”). The continuum reflects a shift from inflammation to pathological bone formation, leading to irreversible structural damage and clinical impairment.

When we discuss the management of the AS, it is noteworthy that the pivotal driver of disability is not merely synovitis or enthesitis but irreversible structural damage resulting from heterotopic ossification, which eventually leads to fusion of the sacroiliac joints and spine (13). (see **Figure 1**).

Patients with axSpA who continue to exhibit high disease activity require treatment strategies primarily based on biologic disease-modifying antirheumatic drugs (bDMARDs). A notable real-world example comes from Lairid et al., who reported that golimumab (GOL) showed superior retention compared with other subcutaneous TNF inhibitors (TNFi) in axSpA, with a mean duration of 59 months, underscoring its clinical utility, particularly in first-line treatment (14). Factors associated with improved persistence on TNFi include male sex, the absence of peripheral manifestations, and earlier initiation of therapy (15). While TNFi are well documented for their efficacy in suppressing both clinical symptoms and inflammatory activity, radiographic progression—particularly the formation of syndesmophytes and ankylosis—may continue (16). Emerging evidence suggests that long-term, sustained TNF inhibition may help mitigate structural progression (17). In addition, recent research over the past decade has demonstrated the promising clinical effects of IL-17A inhibitors, indicating that these small molecules have the potential to significantly improve clinical symptoms in patients with AS, without notable adverse effects or infections (18). It is worth noting that since July 2016, IL-17A inhibitors have been approved for marketing in France (14). In 2025, Lopalco et al. published their insights, highlighting a cytokine-mediated imbalance in axSpA, in which TNFα and IL-17A promote bone erosion via receptor activator of nuclear factor κB ligand (RANKL) and impair osteoblastic bone formation through WNT/β-catenin inhibition. However, they found that no current IL-17A inhibitors have been shown to completely halt or reverse radiographic damage in patients with AS (19). This presents a clinical paradox: targeted biologic therapies effectively suppress clinical and biochemical measures of inflammation, yet their impact on halting radiographic progression is often partial and unpredictable. Recent advancements in understanding the molecular mechanisms of new bone formation in AS have been made; however, key aspects remain unclear. While TNFi have improved treatment outcomes, their efficacy in slowing structural damage is debated, whereas newer biologics targeting the IL-17A/IL-23 axis show promise but require further investigation for long-term effects (20). These results challenge a linear model in which inflammation directly fuels bone formation, underscoring the existence of distinct yet interlinked pathogenic pathways.

**Figure 2:**
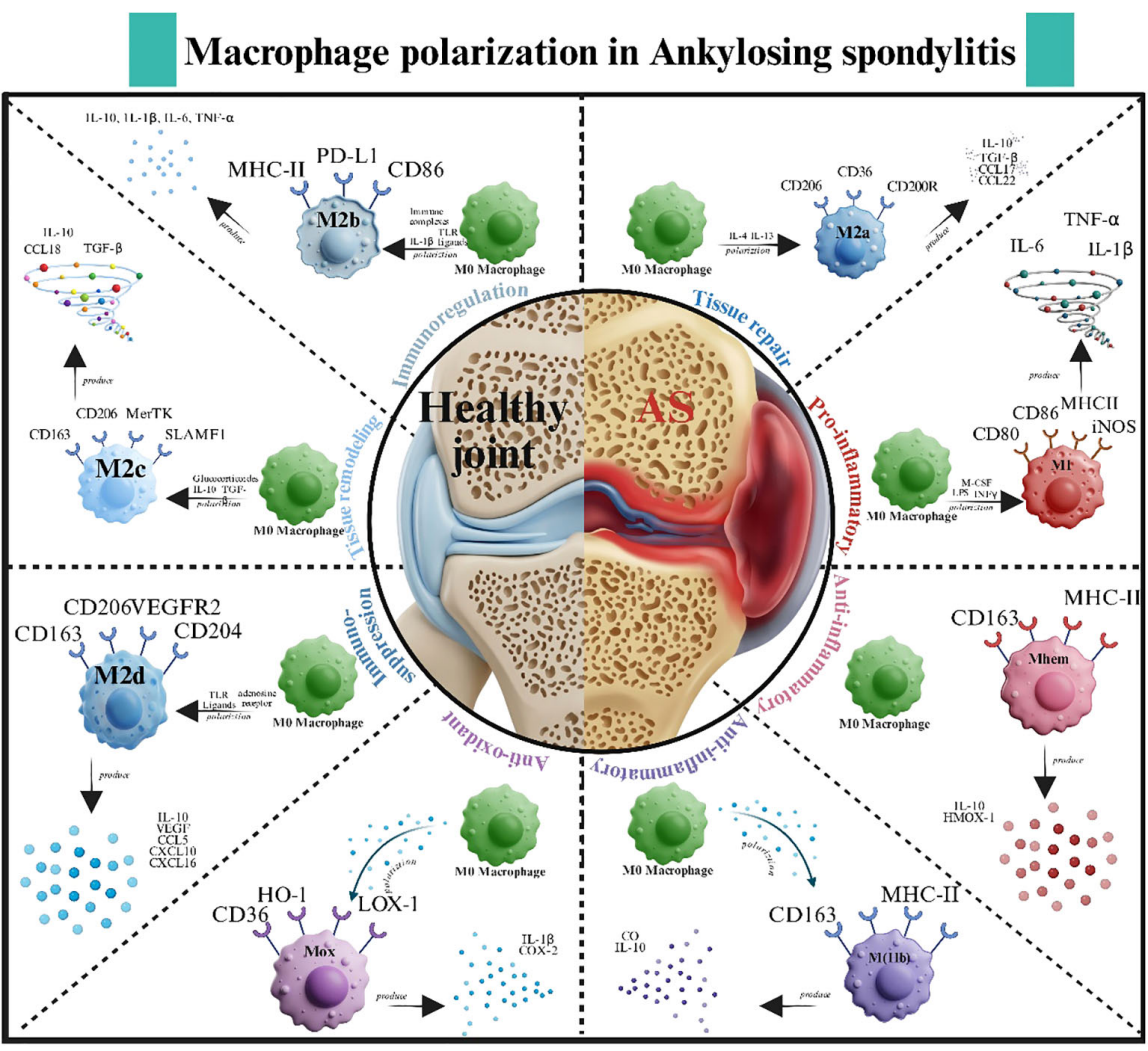
Schematic representation of macrophage polarization in the pathogenesis of ankylosing spondylitis. This figure depicts the functional states of macrophages in the inflammatory microenvironment of AS.

The aforementioned research findings prompted researchers to reconsider their approach, shifting toward an immunological perspective to modulate the aberrant bone formation underlying ankylosing spondylitis, thereby improving disease progression. Current studies have found that the interaction between macrophage-secreted cytokines and other counterparts in the unique entheseal microenvironment may play a crucial role in the pathogenesis of AS (21). Recently proposed by Zhang K et al., monocyte-derived macrophages constitute an essential component of inflammatory responses and are crucial for the activation of autoinflammatory rheumatic diseases (AIRDs) (22). As the primary immune responders within the joint microenvironment, macrophages are the earliest and most abundant immune cells present in the affected synovium (23). They drive disease progression through various mechanisms, including the secretion of pro-inflammatory cytokines, chemokines, and tissue-destructive metalloproteinases (24). The interaction of these cytokines with their corresponding receptors on the surface of different cell types activates several intracellular signaling pathways associated with inflammation and ossification, notably those mediated by extracellular signal-regulated kinases 1 and 2 (ERK1/2), c-Jun N-terminal kinases (JNK), p38 mitogen-activated protein kinases (p38 MAPK), signal transducers and activators of transcription (STATs), and Janus kinases (JAK) (25). The well-documented synergistic effect of IL-17A and TNF-α enhances macrophage production of pro-inflammatory mediators, including IL-6, IL-1β, and matrix metalloproteinases (MMPs), thereby facilitating the transition from early inflammation to chronic arthritis (26). Macrophages exhibit significant plasticity, enabling them to adopt diverse phenotypes and generate subpopulations. This characteristic highlights their dual role in both perpetuating and resolving inflammation, which is heavily influenced by their activation status: classically activated macrophages (M1) or activated macrophages (M2) (27). It is becoming increasingly evident that M1 and M2 phenotypes represent the extremes of a spectrum of macrophage activation (28). This spectrum encompasses “intermediate” phenotypes (M2a, M2b, and M2c, etc) that play crucial roles in immunoregulation and tissue repair and are distinguished by distinct metabolic pathways, surface markers, and cytokine production profiles during AS (29). (**Figure 2**). Within this paradigm, tissue-resident and recruited macrophages emerge as master regulators, exhibiting remarkable plasticity to adopt diverse functional states in response to the local microenvironment (30). Through an extensive review of the prior literature, we found that macrophages are the pivotal “signal integrators” at the interface between inflammation and bone formation in AS. Furthermore, the transforming growth factor-beta (TGF-β) and bone morphogenetic protein (BMP) signaling axis, under the control of these macrophages, is a critical effector pathway responsible for pathological ossification. This review aims to synthesize current evidence into a coherent model: macrophages, polarized by the AS microenvironment, drive pathological spinal fusion through the sustained local production and activation of TGF-β/BMP signaling, which, in turn, orchestrates a recapitulation of endochondral ossification at entheseal sites.

### 1.1 The clinical conundrum of AS – dissociating inflammation from structural damage

Over the past decades, advancements in imaging techniques and therapeutic options have markedly transformed the management of AS (31, 32). However, the clinical course of AS poses a formidable therapeutic challenge, arising from an aberrant immune response in which stromal cells of the sacroiliac joints and spine are misidentified as foreign, resulting in chronic inflammation, severe pain, and reduced mobility. The disease exhibits dual pathological features: a dynamic, immune-driven inflammatory process and progressive structural changes, especially pathological ossification. During the early to moderate stages, the former—manifesting as sacroiliitis, enthesitis, and spinal inflammation—is the primary source of pain and functional impairment; in contrast, the latter ultimately leads to spinal ankylosis, which determines long-term functional decline (33–35).

The advent of bDMARDs targeting key cytokines such as TNF-α and IL-17A has revolutionized treatment, achieving robust and sustained suppression of clinical inflammation and acute-phase reactants (36, 37). Pavelka et al. demonstrated that secukinumab (300 mg) yielded a rapid and significant clinical response—with an ASAS20 rate of 60.5% at week 16—and sustained efficacy through 52 weeks in patients with active AS. However, long-term observational outcomes reveal a substantial proportion of patients continue to exhibit radiographic progression despite achieving clinical remission or low disease activity (38). Although inflammation is controlled, the insidious formation of syndesmophytes and ongoing spinal fusion persists (39). This contradiction underscores the fundamental limitations of current inflammation-centered therapeutic paradigms. It strongly supports the existence of parallel or downstream pathological pathways that are partially decoupled from the initial inflammatory triggers. This insight has prompted a paradigm shift in treatment strategies, underscoring an urgent need for disease-modifying interventions that not only suppress aberrant immune activation but also directly inhibit the pathological osteogenic programs responsible for irreversible structural damage (40).

### 1.2 The AS entheseal niche: a macrophage polarization cauldron

The anatomical predisposition of specific sites—particularly the sacroiliac joints and spinal entheses—to pathology in AS is not a random occurrence but a consequence of their unique structural and functional properties (41, 42). These locations represent distinctive “immuno-mechanical” niches where persistent biomechanical stress converges with chronic inflammatory signals to create a microenvironment that potently drives macrophage polarization and subsequent aberrant tissue repair, ultimately leading to pathological ossification (43). As the point of ligament or tendon insertion into bone, the enthesis is fundamentally a transitional zone that dissipates mechanical loads (44). In AS, physiological functions are transformed into pathological mechanisms as repetitive tensile and shear forces induce microdamage in both the entheseal soft tissue and the adjacent bone (45). This mechanical trauma leads to the release of damage-associated molecular patterns (DAMPs), such as high-mobility group box 1 (HMGB1) and S100 proteins, from stressed or dying stromal cells, including fibroblasts, tenocytes, and osteocytes (46). These endogenous alarmins function as potent activators of the innate immune system. They engage pattern recognition receptors (PRRs), notably toll-like receptors (TLRs), on resident and recruited macrophages, initiating a pro-inflammatory cascade (47). This DAMP-TLR axis serves as a critical link, translating physical micro-injury into a sustained immunogenic signal by affecting macrophages, thereby setting the stage for chronic inflammation, ossification, and ultimately spinal fusion in AS.

**Figure 3:**
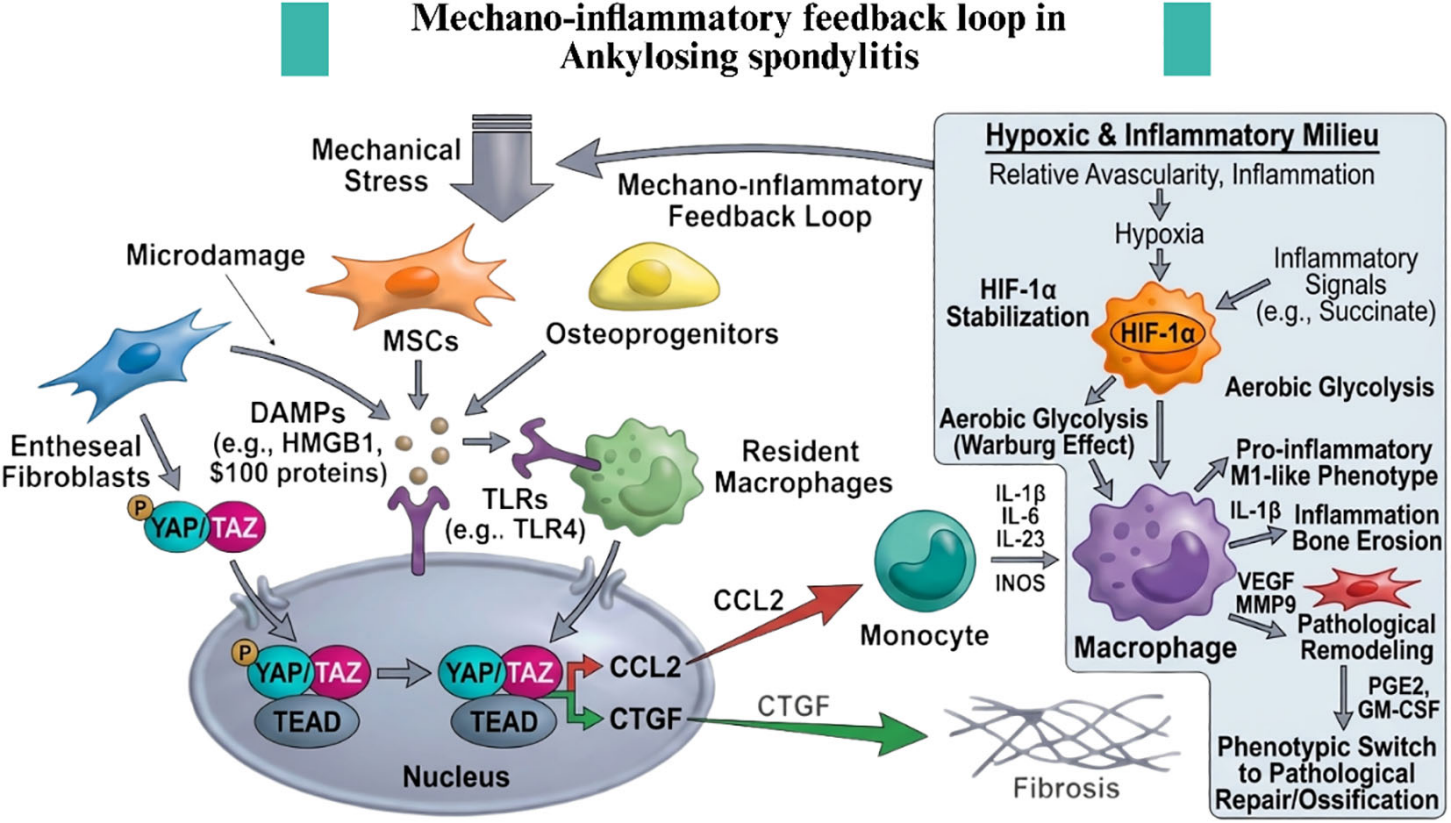
Mechano-inflammatory feedback loop in ankylosing spondylitis. Biomechanical stress induces microdamage at the enthesis, leading to the release of DAMPs that activate LRs. Mechanical signals concurrently activate the YAP/TAZ pathway in entheseal fibroblasts and osteoprogenitors, driving expression of CCL2 and CTGF. Local hypoxia, exacerbated by inflammation, stabilizes HIF-1α and promotes aerobic glycolysis, fostering a pro-inflammatory M1-like macrophage phenotype. These macrophages secrete IL-1β, TNF-α, VEGF, MMP-9, and other mediators that perpetuate inflammation and tissue degradation. The resulting pathological remodeling, together with factors such as PGE2 and GM-CSF, promotes a phenotypic switch toward a pro-osteogenic macrophage state, ultimately driving aberrant ossification and completing a self-amplifying loop that links mechanical injury, inflammation, and structural damage in AS.

Beyond the DAMP-TLR axis, mechanical stress directly reprograms the molecular machinery of local stromal cells. A key mechano-transduction pathway involves the transcriptional co-activators Yes-associated protein (YAP) and transcriptional co-activator with PDZ-binding motif (TAZ) (48). In response to mechanical load, the hippo signaling pathway is inhibited in entheseal fibroblasts, mesenchymal stem cells (MSCs), and osteoprogenitors, leading to dephosphorylation of YAP/TAZ, nuclear translocation, and activation (49, 50). Within the nucleus, YAP/TAZ interacts with the TEA domain transcription factor (TEAD) family to drive the expression of genes involved in cell proliferation, survival, and—crucially—pro-fibrotic and pro-inflammatory mediators (51). These include C-C motif chemokine ligand 2 (CCL2), which recruits monocytes, and connective tissue growth factor (CTGF), which promotes fibrosis (52). On the one hand, the chronic inflammatory milieu further distorts the mechanical microenvironment. Persistent YAP/TAZ activation at these stressed sites is believed to push the normal reparative response into a maladaptive, excessive state, promoting the formation of fibrotic tissue that serves as a scaffold for subsequent endochondral ossification, thus contributing directly to joint ankylosis (53). This mechano-inflammatory feedback loop creates a self-reinforcing niche that continually stimulates stromal cells to produce signals that recruit and educate macrophages. On the other hand, the entheseal microenvironment is further characterized by relative avascularity, a feature exacerbated by inflammation and tissue remodeling. This creates a hypoxic milieu, which is stabilized by inflammation-driven metabolic shifts (54). Hypoxia-inducible factor-1α (HIF-1α) is a master regulator of the cellular response to low oxygen (55). In macrophages, HIF-1α stabilization, driven by both hypoxia and inflammatory signals such as succinate, orchestrates a metabolic switch toward aerobic glycolysis, reminiscent of the Warburg effect observed in cancer cells (56–58). This glycolytic metabolic reprogramming is intrinsically linked to a pro-inflammatory, M1-like phenotype, enhancing macrophage survival, cytokine production (e.g., IL-1β), and functional capacity under adverse conditions (56). Simultaneously, HIF-1α transactivates genes involved in angiogenesis (e.g., VEGF), matrix degradation (e.g., MMP9), and glycolytic enzymes, tailoring macrophage function toward tissue remodeling—a process that, in the context of AS, becomes dysregulated and contributes to pathological repair rather than resolution (59). Therefore, hypoxia acts not merely as a bystander condition but as an active instructor of macrophage phenotype and function within the entheseal niche. (**Figure 3**).

The initial immune dysregulation characteristic of AS floods the local entheseal environment with a specific cytokine cocktail. Key cytokines include IL-17A, TNF-α, and IL-23, which are produced by entheseal-resident immune cells, including type 3 innate lymphoid cells (ILC3s), γδ T cells, and Th17 cells (60). These cytokines orchestrate a dominant shift toward classical M1-like macrophage polarization. This phenotype is characterized by elevated production of IL-1β, IL-6, and IL-23, as well as inducible nitric oxide synthase (iNOS), which contributes to local tissue inflammation and promotes osteoclast-mediated bone erosion at inflammatory sites (61). However, this acute inflammatory phase is paradoxically instrumental in setting the stage for later ossification. Notably, TNF-α and IL-17A can stimulate stromal cells to secrete mediators that modulate macrophage plasticity, including prostaglandin E2 (PGE2) and granulocyte-macrophage colony-stimulating factor (GM-CSF) (62, 63). These factors may prime macrophages for a subsequent phenotypic switch, bridging the acute inflammatory response to a chronic reparative—and ultimately pathological—bone-formation state (63).

In summary, the AS enthesis is far more than a passive site of inflammation. It is a highly specialized and dynamic “polarization cauldron” where biomechanical stress, hypoxic conditions, and a distinct inflammatory cytokine milieu intersect. This triad of signals—mechanical (via DAMPs/YAP/TAZ), metabolic (via hypoxia/HIF-1α), and immunological (via IL-17A/TNF-α/IL-23)—actively converges to shape macrophage recruitment, survival, and functional polarization. The initial pro-inflammatory (M1-like) response, while driving symptoms, also remodels the niche and primes macrophages for a fateful transition. As explored in the following sections, this unique microenvironment fosters the emergence of macrophage subsets that are equipped not only to sustain inflammation but also to become pivotal architects of the pathological osteogenic program through the TGF-β/BMP signaling axis.

### 1.3 Macrophage polarization: a spectrum of pro-osteogenic phenotypes in AS

The functional plasticity of macrophages, enabling their adaptation to a spectrum of activation states beyond the classical M1/M2 dichotomy, is important to the pathophysiology of AS (64, 65). During the transition from an inflammatory to an ossifying phenotype, macrophages adopt distinct, disease-specific profiles that drive pathological bone formation (66). Recent single-cell transcriptomic analyses have revolutionized our understanding of macrophage heterogeneity in synovial tissues (67, 68). In spondyloarthritis, specific macrophage subsets are markedly enriched at sites of enthesitis and new bone formation. These subsets often express markers such as triggering receptor expressed on myeloid cells 2 (TREM2), secreted phosphoprotein 1 (SPP1/osteopontin), the hemoglobin scavenger receptor CD163, and the mannose receptor CD206 (69). Critically, many of these cells exhibit a hybrid transcriptional signature, co-expressing genes associated with both inflammatory responses (e.g., IL1B, TNF-α) and tissue remodeling (e.g., TGF-β1, VEGFA), indicating their role as integrators of pro-inflammatory and pro-reparative signals (70). (**Table 1**).

**Table 1:** Macrophage polarization phenotypes and TGF-β/BMP signaling in AS.

Macrophage phenotypes	Markers	Inducer	Related cytokine	Functions & relevance in AS	Refs.
Pro-inflammatory phenotypes					
M1-like (classically activated)	CD80, CD86, MHC-II, TLR-2/4, iNOS	IFN-γ, LPS, TNF	TNF-α, IL-1β, IL-6, IL-12, IL-23, NO, ROS	Drives spinal inflammation, promotes osteoclastogenesis, correlates with active sacroiliitis, and syndesmophyte formation.	(71, 72)
Osteoclastogenic macrophages	CX3CR1^+^, CD14^+^, CD64^+^, RANK^+^	RANKL, TNF-α, IL-1β, GM-CSF	TNF-α, IL-1β, IL-6, IL-12, IL-23, NO, ROS	Directly contributes to bone erosion and focal osteolysis in AS; enhanced by pro-inflammatory cytokine milieu	(73)
Anti-inflammatory/pro-fibrotic phenotypes					
M2a (alternatively activated)	CD206, IL-1R, ARG1, FIZZ1	IL-4, IL-13	IL-10, TGF-β, CCL17/18/22	Promotes tissue repair and fibrotic responses; may contribute to entheseal fibrosis and ankylosis in late AS	(74, 75)
M2c (deactivated)	CD163, CD206, MerTK, ARG1	IL-10, glucocorticoids	IL-10, TGF-β, CCL16/18	Associated with inflammatory resolution; may modulate but not fully suppress ankylosis in AS.	(76)
Intermediate/hybrid phenotypes					
Hybrid M1–M2	Co-expression of CD80, CD163, CD206, TLR4	Mixed cytokines (e.g., IFN-γ + IL-10)	TNF-α, IL-10, TGF-β, IL-6, GM-CSF	Present in chronic enthesitis; may simultaneously drive inflammation and tissue remodeling, linking inflammation to bone formation in AS	(77, 78)
TGF-β/BMP-associated macrophages					
TGF-β–polarized macrophages	CD163, CD206, TGFBR1/2	TGF-β1, BMP-2, mechanical stress	TGF-β1, IL-10, PDGF, VEGF	Key role in osteoblast differentiation, syndesmophyte formation, and ligamentous ossification via SMAD signaling	(79, 80)
BMP-responsive macrophages	ALK2/3, BMPR1/2, pSMAD1/5/9	BMP-2, BMP-7, inflammatory stimuli	BMP-2, BMP-7, TNF-α, IL-6	Enhance osteogenic differentiation in entheses; implicated in pathological bone formation in AS	(81, 82)

Chen J. et al. found that the intermediate monocyte subset (CD14++CD16+), a key precursor of tissue macrophages, is expanded in the peripheral blood and synovial fluid of patients with AS and exhibits a pre-activated, pro-inflammatory phenotype (83). Upon recruitment to the entheseal niche, these cells are exposed to a unique confluence of signals—mechanical stress, hypoxia, and a cytokine milieu rich in IL-17A, TNF-α, and IL-23—which collectively shape their polarization (84). Initially, this environment promotes a classical M1-like activation, characterized by the production of IL-1β, IL-6, and IL-23, as well as iNOS, which contributes to lo cal inflammation and osteoclast-mediated bone erosion (85). However, this inflammatory phase is not terminal; it sets the stage for a phenotypic shift. As acute inflammation subsides, either spontaneously or due to therapy, the microenvironment evolves. The clearance of apoptotic cells (efferocytosis), rising levels of IL-10, and the increasing presence of latent TGF-β and immune complexes drive macrophages toward an M2-like or “alternatively activated” phenotype (86, 87). This transition involves downregulation of pro-inflammatory mediators and upregulation of factors involved in matrix deposition, angiogenesis, and crucially, osteogenesis (88).

Of paramount importance in AS is the emergence of macrophage subsets with explicit pro-osteogenic capacity (89). Transcriptomic studies of AS synovial and entheseal tissues reveal macrophage populations with high expression of potent osteogenic cytokines, particularly TGF-β1 and BMP2 (90). These macrophages are strategically localized at the advancing front of ossifying lesions. The pro-osteogenic function of these cells is not merely a passive secretory role but involves active engineering of the local niche. As a significant source of latent TGF-β complexes and various BMPs (e.g., BMP2, BMP4, BMP7) (91, 92), macrophages also express integrins (αvβ6, αvβ8) and secrete proteases such as MMP-2, MMP-9, and MMP-13, and play a pivotal role in activating latent TGF-β stored in the ECM, thereby generating a potent and localized osteogenic signal gradient (93, 94). This process is likely amplified by the hypoxic conditions at entheses, which stabilize HIF-1α and promote a glycolytic metabolic state in macrophages, a metabolic profile linked to both sustained inflammatory signaling and tissue remodeling responses (95). In 2025, experts from Sun Yat-sen University (Hao et al.) revealed that TREM2^+^ macrophages, induced by IL-33, promote pathological new bone formation in AS by secreting CREG1 to activate the IGF2R-PI3K-AKT pathway in ligament-derived progenitor cells (96). Macrophage presence in entheseal lesions suggests a potential link between resolving inflammation, clearance of tissue debris, and the initiation of a fibro-ossific repair program. Research by Schlundt et al. underscores that a fine balance in M1/M2 macrophage function is essential for effective fracture healing, as macrophage depletion perturbs endochondral ossification, whereas therapeutic skewing toward the M2 phenotype is a viable strategy to augment bone repair (97).

Therefore, macrophage polarization in AS represents a dynamic continuum in which cells can adopt a spectrum of pro-osteogenic phenotypes. These phenotypes are characterized by co-expression of immunoregulatory and osteogenic molecules, enabling them to respond to and perpetuate abnormal repair signals within the entheseal microenvironment. Polarization is driven by an interplay among biomechanical stress (via DAMPs and YAP/TAZ activation), hypoxia (via HIF-1α), and specific cytokine networks (IL-17A, TNF-α, TGF-β). This plasticity enables macrophages to transition from promoting inflammation to orchestrating pathological tissue repair, ultimately leading to the aberrant endochondral ossification characteristic of syndesmophyte formation and spinal fusion. Targeting these specific pro-osteogenic macrophage subsets or the signals that generate them, rather than viewing macrophages as a homogeneous population, presents a promising therapeutic strategy to uncouple inflammation from structural progression in AS.

### 1.4 Overview of TGF-β and BMP signaling pathways in AS

In the 1970s, TGF-βs were initially identified for their ability to induce phenotypic transformation in fibroblasts (98). Almost simultaneously, BMPs were discovered for their remarkable capacity to induce ectopic bone and cartilage formation (99). These cytokines are synthesized as large precursor proteins that undergo proteolytic cleavage. The mature homodimeric ligands remain non-covalently associated with their prodomain, forming a latent complex that is sequestered in the ECM or on the cell surface (100, 101). This latency is a critical regulatory checkpoint, as the biological activity of these potent morphogens is tightly controlled at the level of ligand activation, a process in which immune cells, such as macrophages, are deeply involved. Chaikuad A.’s 2016 research showed that the TGF-β superfamily comprises a critical group of secreted cytokines that regulate a diverse array of cellular and physiological processes, including cell proliferation, differentiation, apoptosis, migration, and ECM production (102). Within this superfamily, the TGF-β/Activin/Nodal and BMP/growth and differentiation factor (GDF) subfamilies are particularly significant in the context of skeletal biology (103, 104). Wu M et al. pointed out that the TGF-β and BMP signaling pathways associated with these subfamilies are not only essential for embryonic development and the maintenance of postnatal tissue homeostasis but also play a central role in the pathogenesis of various skeletal disorders (105). Therefore, a comprehensive understanding of the intricate mechanisms underlying TGF-β and BMP signaling may be vital for unraveling the molecular intersections through which inflammation contributes to pathological bone formation in AS.

Signal transduction is initiated when a mature, active TGF-β or BMP ligand binds to a specific set of transmembrane serine/threonine kinase receptors (106). These receptors fall into two types: Type I (also termed activin receptor-like kinases, ALKs) and Type II. The canonical assembly forms a tetrameric complex comprising two Type I and two Type II receptors (107–109). Typically, the ligand first binds to the constitutively active Type II receptor, which then recruits and transphosphorylates the Type I receptor’s glycine-serine-rich (GS) domain (110). This phosphorylation event activates the Type I receptor kinase, which serves as the gateway for downstream intracellular signaling (111). The primary and most extensively studied downstream effectors are the receptor-regulated Smads (R-Smads) (112). The ligand-receptor complex determines which R-Smad pathway is engaged: TGF-βs and activins generally signal through Smad2 and Smad3, whereas BMPs, GDFs, and certain TGF-β isoforms signal primarily through Smad1, Smad5, and Smad8 (also known as Smad9) (113). Upon phosphorylation by the activated Type I receptor, R-Smads form a heterotrimeric complex with the common mediator Smad (Co-Smad), Smad4 (114). Subsequently, this complex translocates into the nucleus, where it interacts with a variety of DNA-binding cofactors, co-activators, and co-repressors to modulate the transcription of target genes (115). This Smad-dependent pathway regulates the transcription of genes pivotal to cell fate determination, including the osteogenic master regulator RUNX2 and the chondrogenic transcription factor SOX9, which are essential in endochondral ossification and are recapitulated in AS syndesmophytes (116).

However, TGF-β/BMP signaling pathways are highly complex and context-dependent, exhibiting significant signal diversification through multiple noncanonical, Smad-independent mechanisms (117). Activated receptor complexes can also trigger diverse intracellular signaling pathways, including the MAPK cascades—p38, JNK, and ERK—along with the phosphatidylinositol 3-kinase (PI3K)/Akt pathway and small GTPases such as Rho and Rac (118). These signaling pathways frequently interact with or regulate the Smad pathways, ensuring precise modulation of cellular responses. For example, p38 MAPK can phosphorylate and stabilize RUNX2, enhancing the osteogenic transcriptional program activated by BMP-Smad signaling (119). Furthermore, the temporal dynamics, amplitude, and spatial confinement of the signal are precisely modulated by a variety of extracellular and intracellular regulators. Extracellular antagonists, including Noggin, Chordin, and Gremlin, interact directly with BMP ligands, inhibiting receptor binding (120). Intracellular inhibitory Smads (I-Smads), specifically Smad6 and Smad7, competitively inhibit receptor binding of R-Smads or facilitate receptor degradation, thereby constituting a vital negative feedback mechanism (121). The divergent and occasionally antagonistic biological effects of TGF-β and BMP signaling are fundamental to their functions in bone metabolism. BMP signaling predominantly promotes osteoblast differentiation and osteogenesis, whereas TGF-β signaling exhibits a more nuanced, stage-dependent role (122, 123). During initial phases, TGF-β facilitates the recruitment and proliferation of MSCs and pre-osteoblasts. However, it also exerts inhibitory effects on terminal osteoblast differentiation and mineralization, while serving as a pivotal mediator of fibrotic processes that often precede ossification (124). This duality is evident in receptor utilization: BMPs predominantly signal through ALK1, ALK2, ALK3, and ALK6, whereas TGF-βs primarily signal through ALK5 (TGFβRI). The interplay and regulation between these pathways, modulated by the local microenvironment, ultimately influence whether physiological repair proceeds or pathological heterotopic ossification occurs (125).

In AS, dysregulation of the TGF-β/BMP signaling axis is a fundamental aspect of disease pathogenesis (24). On the one hand, genetic variations in genes involved in this pathway have been linked to increased susceptibility. On the other hand, animal models with impaired TGF-β/BMP signaling display skeletal characteristics that closely mirror those seen in human spondyloarthritis (126). As detailed in the sections above, the unique inflammatory and mechanical microenvironment of the AS enthesis shapes macrophage behavior, which, in turn, becomes a dominant local source and activator of TGF-βs and BMPs. This creates a self-amplifying niche that hijacks these evolutionarily conserved developmental pathways, driving the erroneous endochondral ossification that leads to spinal fusion.

Beyond Smad-dependent signaling, cross-talk with other key pathways adds further complexity to the osteogenic program in AS. The Wnt/β-catenin pathway, a central regulator of bone formation, exhibits reciprocal interactions with TGF-β/BMP signaling: while BMPs can synergize with Wnt to promote osteoblast differentiation, excessive TGF-β may antagonize Wnt activity, contributing to the uncoupling between inflammation and bone formation (122, 123). Furthermore, emerging evidence highlights the role of epigenetic regulation in macrophage polarization and osteogenesis. For instance, microRNA-21 (miR-21) has been shown to modulate TGF-β/Smad7 signaling and promote fibrotic and osteogenic responses, and its inhibition reduces entheseal new bone formation in preclinical models (**Table 2**). Similarly, N6-methyladenosine (m6A) RNA modification—exemplified by METTL3-mediated methylation—has been implicated in macrophage phenotypic switching and may represent a novel regulator of the immuno-osteogenic axis in AS (138). These intersecting pathways and epigenetic mechanisms offer additional therapeutic opportunities beyond canonical Smad signaling.

**Table 2:** Current therapeutics available or under trial for ankylosing spondylitis targeting macrophage polarization phenotypes and TGF-β/BMP signaling.

Agent/intervention	Mechanism of action	Target phenotype/pathway	Clinical/preclinical outcomes	Refer.
TNFi (e.g., Adalimumab, Infliximab)	Suppress TNF-α signaling, reduce inflammatory cytokine production	M1 polarization ↓, osteoclastogenesis ↓	Reduce inflammation, improve symptoms, slow radiographic progression in AS	(127, 128)
IL-17 inhibitors (e.g., Secukinumab, Ixekizumab)	Neutralize IL-17A, attenuate Th17-driven inflammation	M1 polarization ↓, BMP pathway modulation	Significant clinical improvement, reduction in spinal inflammation	(129, 130)
JAK inhibitors (e.g., Tofacitinib)	Inhibit JAK/STAT signaling, modulate cytokine production	M1/M2 balance modulation, TGF-β signaling ↓	Reduced disease activity, ongoing trials for AS	(131)
TGF-β pathway modulators (e.g., Fresolimumab)	Neutralize TGF-β1/2/3, inhibit SMAD2/3 signaling	TGF-β-driven fibrosis & osteogenesis ↓	Preclinical evidence in entheseal ossification models	(132)
BMP signaling inhibitors (e.g., LDN-193189)	Inhibit ALK2/3, suppress BMP-SMAD1/5/8 pathway	BMP-driven osteogenesis ↓	Reduced syndesmophyte formation in animal models	(133–135)
Mesenchymal stem cell (MSC) therapy	Paracrine modulation of macrophages, promote M2 polarization	M2 polarization ↑, TGF-β/BMP balance modulation	Anti-inflammatory & osteogenic modulation in preclinical AS models	(136, 137)
Anti-miR-21 oligonucleotides	Downregulate miR-21, attenuate TGF-β/SMAD7 signaling	Fibrotic & osteogenic responses ↓	Reduced entheseal new bone formation in mice	(138)
Nanoparticle-loaded dexamethasone	Targeted delivery to entheseal macrophages, sustained anti-inflammatory effect	M1 polarization ↓, local inflammation ↓	Enhanced therapeutic efficacy, reduced systemic side effects in preclinical studies	(139)
BMP-2/7 trap proteins (nanocarrier)	Sequester BMP ligands, inhibit pathological osteogenesis	BMP signaling ↓	Suppression of heterotopic ossification in AS models	(140)
TNFi + BMP inhibitor (e.g., ABP 501 (adalimumab-atto))	Dual suppression of inflammation and osteogenesis	M1 ↓ & BMP pathway ↓	Synergistic reduction in inflammation and new bone formation in preclinical models	(141)

A downward arrow (↓) denotes suppression, downregulation, or attenuation of the indicated parameter. An upward arrow (↑) signifies enhancement, upregulation, or promotion of the parameter.

### 1.5 Macrophages as engineers of the TGF-β/BMP signaling transduction

In addition to their established functions in immunological defense and phagocytic clearance, macrophages serve as key orchestrators of tissue homeostasis and regenerative processes, functioning as dynamic modulators of the local signaling microenvironment (142, 143). Within the realm of skeletal biology, a critical aspect of this engineering function is the capacity to precisely regulate the activation and bioavailability of two pivotal cytokine families: TGF-β and BMPs (144). These cytokines are produced and secreted as latent complexes that necessitate specific activation mechanisms to attain biological activity. Macrophages, especially osteoclasts, are now acknowledged as key regulators of this activation process, thereby critically influencing TGF-β and BMP signaling pathways within skeletal tissues (145). The latent TGF-β complex, consisting of the mature cytokine non-covalently associated with its latency-associated peptide (LAP), is stored within the bone ECM. This reservoir is often stabilized by the binding of latent TGF-β-binding proteins (LTBPs), which anchor the complex to structural ECM components such as fibrillin. The release and activation of TGF-β from this ECM pool are critically dependent on osteoclastic resorption activity (146). During bone resorption, osteoclasts generate an acidic microenvironment within the resorption lacuna via the V-ATPase proton pump. This acidic pH promotes the dissociation of the mature TGF-β dimer from its latent complex (147). Furthermore, osteoclasts secrete proteolytic enzymes, particularly MMPs such as MMP-9 and MMP-13, which can cleave LAP and LTBP, thereby liberating active TGF-β (148). Niikura K’s findings supported this mechanistic pathway that genetic knockout of the Atp6i gene, which encodes an essential subunit of the osteoclastic V-ATPase complex, impairs osteoclast function and prevents TGF-β activation in bone tissue (149). Thus, osteoclasts are not merely bone destroyers but are essential “switches” that convert stored, inactive TGF-β into a potent, localized signaling molecule (150). In 2019, Swedish experts Lerner UH et al. found that this released TGF-β can act in a paracrine manner to recruit MSCs and osteoprogenitors to the resorption site, coupling bone resorption to subsequent bone formation (151).

A recent study from researchers at Central South University demonstrated that the bioavailability of particular BMPs is also modulated by proteolytic activity originating from bone-resident macrophages (BMMs) (152). Subsequent structural analyses demonstrated that certain BMPs, including BMP4 and BMP7, are not kept in a traditional latent state by their pro-domains. Their activation and interactions with ECM components can be influenced by matrix turnover (153). However, other pro-BMPs are often processed into their active forms through proteolytic cleavage by enzymes, including MMPs and members of the a disintegrin and metalloproteinase with thrombospondin motifs (ADAMTS) family (154). Because macrophages and osteoclasts are rich sources of these proteases, they are strategically positioned to regulate local concentrations of active BMP ligands. For instance, BMP2, a critical osteoinductive factor, is embedded within the mineralized bone matrix (155). Osteoclastic resorption not only releases mineral and matrix-bound factors but may also process BMP precursors, thereby contributing to the pool of active BMPs available during bone remodeling and fracture healing (156). Moreover, macrophages can influence the TGF-β/BMP signaling landscape by modulating the expression of endogenous antagonists and co-receptors (157). The inflammatory activation of macrophages can influence their secretion of BMP antagonists such as Noggin and Gremlin-1. Specifically, M1-like macrophages may promote an antagonistic environment that inhibits BMP-driven osteogenesis, while M2-like macrophages tend to decrease antagonist expression, thereby enhancing BMP signaling and supporting tissue regeneration (158). Although this specific regulatory axis requires further direct investigation in skeletal contexts, the paradigm is well-established in other regenerative processes. Significantly, the engineering role of macrophages extends beyond ligand activation to include the clearance of signaling components (159). Through phagocytosis, macrophages can remove cellular debris and apoptotic bodies that may contain signaling receptors, ligands, or inhibitors, thereby resetting the tissue microenvironment’s signaling potential (160). This clearance function is vital during the resolution phase of inflammation and repair, ensuring that transient signaling events are appropriately terminated. In summary, macrophages, particularly osteoclasts, function as essential modulators of the TGF-β/BMP signaling environment in bone tissue. During resorption, they utilize targeted acidification and protease secretion to activate matrix-bound TGF-β and are likely involved in BMP activation as well. This positions them as key regulators of the coupling mechanism between bone resorption and formation. Additionally, their secretory and phagocytic capabilities enable them to modulate the extracellular milieu by influencing the distribution of signaling agonists, antagonists, and pathway modulators.

### 1.6 TGF-β/BMP signaling—the osteogenic execution program in AS

The pathological osteogenesis in AS shares fundamental molecular and cellular mechanisms with physiological bone formation and repair, with the TGF-β superfamily, particularly the BMP subfamily, serving as the central executor of the osteogenic program (161). While inflammatory triggers (e.g., IL-17/23) set the stage, it is the dysregulated, sustained activation of TGF-β/BMP signaling that directly drives the differentiation of mesenchymal progenitor cells into osteoblasts and orchestrates aberrant bone formation at entheseal sites and within joints (162). Recent studies have confirmed that the mammalian genome encodes 33 TGF-β family ligands, which can be broadly categorized by their receptor usage and downstream Smad signaling pathways (163). BMPs (such as BMP-2, -4, -6, -7, 9) and GDFs (e.g., GDF-5, -6, 7) primarily signal through specific type I receptors (ALK-1, -2, -3, -6) and type II receptors (e.g., BMPRII, ActRIIA/B), leading to the phosphorylation and activation of receptor-regulated Smads (R-Smads) Smad1, Smad5, and Smad8. These activated R-Smads then form complexes with the common mediator Smad4 (164). The complexes translocate into the nucleus, where they cooperate with lineage-specific transcription factors, chromatin modifiers, and other co-regulators to activate the transcription of osteogenic target genes (165). Key targets include RUNX2 (a master transcription factor for osteoblast differentiation), osterix (SP7), alkaline phosphatase (ALPL), and various bone matrix proteins like osteocalcin (BGLAP) and type I collagen (COL1A1) (166, 167). In the context of AS, multiple lines of evidence indicate a crucial role for hyperactive BMP signaling. Histological studies have demonstrated that at sites of enthesitis and syndesmophyte formation, expression of BMPs and phosphorylated Smad1/5/8 is upregulated, indicating that the canonical BMP-Smad1/5/8 pathway serves as a potent inducer of osteoblast differentiation and bone formation (168). Genetic association studies, while not directly implicating BMP genes as key loci for AS susceptibility, have elucidated pathways involved in bone formation and remodeling processes. More directly, animal models of spondyloarthritis have demonstrated that BMP signaling is essential for pathologic osteoproliferation. Inhibition of BMP activity, either by administering endogenous antagonists such as Noggin or by using small-molecule inhibitors of BMP type I receptors, can significantly reduce or even prevent ectopic bone formation in these models without completely halting inflammation (169). This underscores that inflammation creates a permissive environment, whereas BMP signaling executes the osteogenic program.

While BMPs are the primary osteogenic drivers, TGF-β itself (particularly TGF-β1) plays a complex and context-dependent role in this process, which may be equally critical in AS. TGF-βs signal through distinct receptors (TβRI/ALK-5 and TβRII), leading to activation of Smad2 and Smad3 (170). In the early stages of MSC commitment, TGF-β signaling can inhibit terminal osteoblast differentiation and bone mineralization by suppressing RUNX2 activity and promoting maintenance of a progenitor state; however, in the context of a pro-fibrotic and pro-osteogenic microenvironment—exactly what exists in the inflamed enthesis—TGF-β’s role shifts (171). TGF-β1 is a potent inducer of fibrosis by stimulating the production of ECM components like collagen and fibronectin, and by promoting the transformation of fibroblasts and other cells into matrix-producing myofibroblasts (172). In AS, this fibrotic response is a key precursor to ossification, as the newly formed fibrocartilaginous tissue serves as the scaffold or “anlage” for subsequent endochondral bone formation (173, 174). Surprisingly, TGF-β can synergize with BMP signaling. It can enhance the recruitment and proliferation of osteoprogenitor cells to the site of the lesion. More importantly, TGF-β-activated Smad3 can interact with BMP-activated Smad1/5/8 and Smad4 complexes, potentially modulating the transcriptional output toward a more robust osteogenic response or stabilizing the pre-osteoblastic phenotype (101, 175). The activation of latent TGF-β in the ECM, often mediated by integrins such as αvβ6 and αvβ8 expressed on activated fibroblasts or inflammatory cells, provides a localized and sustained source of active TGF-β that fuels this fibro-osseous transition (176).

Therefore, in AS, the “osteogenic execution program” is not mediated by a single ligand but is likely the result of a dysregulated signaling network where BMP and TGF-β pathways converge and interact. Inflammatory cytokines (e.g., TNF, IL-17) upregulate the expression of BMPs and TGF-β, as well as their receptors, in resident macrophages and infiltrating MSCs (100, 177). The resulting BMP-Smad1/5/8 signaling initiates and drives the osteoblast differentiation program. Concurrently, TGF-β-Smad2/3 signaling promotes the deposition of a collagen-rich fibrotic matrix and may modulate the BMP response, creating a microenvironment highly conducive to ossification. This aberrant, self-perpetuating osteogenic program ultimately leads to the replacement of ligament and enthescal soft tissues with mature, bridging bone, characteristic of AS progression (178).

## 2 Therapeutic implications: targeting the immuno-osteogenic crossroads of AS

The management of AS has evolved from symptom-focused interventions to strategies targeting the fundamental disease process. Contemporary understanding frames AS as a disorder of the “immuno-osteogenic crossroads, “ where inflammatory dysregulation and pathological bone formation are mechanistically interlinked (17). This paradigm shift demands therapies that move beyond generalized immunosuppression to precisely disrupt the pathogenic crosstalk between immune cells, stromal cells, and osteogenic signaling cascades.

### 2.1 Established and evolving pharmacological strategies

The therapeutic foundation for AS remained mainly NSAIDs, primarily utilized for symptom control. However, ASAS-EULAR recommendations for the management of now suggest a potential disease-modifying effect, particularly for continuous, high-dose NSAID use in established radiographic axial spondyloarthritis (r-axSpA) (179). Analysis from the German Spondyloarthritis Inception Cohort (GESPIC) demonstrated that a 10-point increase in the NSAID intake score was associated with a statistically significant reduction in radiographic spinal progression, with an adjusted β-coefficient of -0.077 (95% CI: -0.152, -0.003) over 2-year intervals in the r-axSpA subgroup, an effect most pronounced with COX-2 inhibitors (180). For patients with persistently elevated disease activity, bDMARDs targeting specific cytokines are the standard escalation. Recently EULAR announced that TNFi and IL-17A inhibitors (IL-17Ai) have established profound efficacy (181). In clinical trials, IL-17Ai such as secukinumab achieved an assessment of the Spondyloarthritis International Society 40% improvement criteria (ASAS40) response rate of approximately 60% versus 29% for placebo at Week 16, alongside significant inhibition of radiographic progression (129). In 2015 Kroon FP et al. published that small-molecule JAK inhibitor (JAKi), exemplified by the selective JAK1 inhibitor ivarmacitinib, have demonstrated rapid and significant efficacy in the treatment of the AS (182). In a Phase II/III trial, ivarmacitinib 4 mg daily yielded an ASAS40 response of 32.1% versus 18.3% for placebo at Week 12, with corresponding statistically significant improvements across multiple patient-reported outcome domains including total back pain VAS (mean change -25.6 vs. -17.0, P < 0.001) and bath AS disease activity index (BASDAI) (-2.3 vs. -1.5, P < 0.05) (183). **Table 2** provides current therapeutics available or under trial for AS targeting macrophage polarization phenotypes and TGF-β/BMP signaling.

Nevertheless, significant unmet needs persist, particularly in fully arresting the pathological osteogenesis (syndesmophytosis) that drives structural damage. This clinical challenge highlights the limitations of targeting isolated inflammatory axes without directly engaging the core osteogenic execution program. Consequently, the next frontier involves developing agents that concurrently modulate the immune microenvironment and directly inhibit osteogenic drivers.

### 2.2 Macrophage polarization: a central regulator at the crossroads

A pivotal and underexplored therapeutic node lies in the polarization state of tissue macrophages, which function as central regulators at the immuno-osteogenic crossroads. These plastic cells exist within distinct macrophage phenotypes, critically shaping the local milieu. In AS, a reciprocal link exists between macrophage polarization and the TGF-β/BMP signaling pathway, a core osteogenic driver. TGF-β is a potent inducer of M2-like macrophages, which in turn can produce more TGF-β and serve as a source of BMPs, thereby fueling a self-reinforcing pro-fibrotic and pro-osteogenic feedback loop. This positions macrophages as strategic master regulators linking inflammation to bone formation, and future therapeutic research on AS should consider this background information.

### 2.3 Emerging biologics targeting the macrophage–TGF-β/BMP axis

The mechanistic framework established in the preceding sections—wherein macrophages serve as both principal sources and local activators of TGF-β/BMP ligands within the AS entheseal niche—directly informs the rational development of next-generation therapeutic strategies. Rather than targeting individual cytokines in isolation, these emerging approaches aim to dismantle the self-amplifying loop that links macrophage polarization to pathological osteogenesis. Consequently, the therapeutic paradigm is shifting from broad-spectrum immunosuppression toward more refined, mechanism-based interventions that concurrently modulate immune cell behavior and the core osteogenic execution program (17).

Several classes of agents currently under preclinical or clinical investigation illustrate this transition (summarized in **Table 2**). IL-23/IL-17A inhibitors and selective JAK1 inhibitors (e.g., ivarmacitinib, tofacitinib) primarily suppress the upstream cytokine milieu that promotes M1-like polarization; however, their downstream effects include attenuation of inflammation−driven BMP expression in stromal cells, thereby indirectly dampening osteogenic signaling (184). A more direct osteo−centric strategy is embodied by BMP ligand inhibitors (e.g., recombinant Noggin, the small−molecule ALK2/3 inhibitor LDN−193189) and ALK−1 inhibitors, which are designed to block the BMP−Smad1/5/8 pathway—the core executioner of ectopic bone formation. Preclinical studies have demonstrated that Noggin administration significantly reduces syndesmophyte formation in animal models of spondyloarthritis (133, 185, 186). Given the pivotal role of TGF−β bioavailability, integrin αvβ6/αvβ8 inhibitors represent a mechanistically strategic intervention. By preventing the localized, cell−tension−dependent activation of latent TGF−β within the ECM, these agents limit the pool of bioactive ligand available to drive both M2−polarization and pathological osteogenesis (189). It is important to note, however, that integrin αvβ6/αvβ8 inhibitors remain largely at the preclinical stage for AS; their evaluation has been primarily confined to models of pulmonary fibrosis and tumor immunity, and direct evidence in spondyloarthritis is currently lacking (189).

Looking toward the next generation of therapeutics, dual−activity or bispecific agents engineered to simultaneously reprogram macrophages and inhibit osteogenic signaling represent a conceptually attractive but still highly exploratory frontier. A hypothetical agent might, for example, deliver M2−polarizing signals (e.g., via IL−4/IL−13 receptor engagement) while sequestering active TGF−β, thereby actively dismantling the pathogenic feedback loop. Such approaches, however, have not yet entered preclinical development for AS and face substantial challenges in target selection, pharmacokinetics, and tissue−specific delivery. Any therapeutic strategy that interferes with the TGF−β/BMP axis must contend with the pleiotropic nature of these pathways. Systemic inhibition of TGF−β signaling has been associated with significant on−target toxicities, including cardiovascular inflammation (e.g., valvular lesions, hemorrhagic gastritis) and impaired wound healing, as observed in clinical trials of the pan−TGF−β neutralizing antibody fresolimumab in cancer and fibrosis (132). Although no such agent has been formally tested in AS, these safety signals underscore the likely need for localized or conditional targeting—for instance, via nanoparticle−mediated delivery to entheseal sites or the use of prodrugs activated specifically within the inflammatory microenvironment—rather than systemic TGF−β blockade. In contrast, BMP−directed inhibitors (e.g., LDN−193189) have demonstrated more favorable safety profiles in preclinical models of heterotopic ossification, though long−term effects on physiological bone remodeling remain to be fully characterized (186). For integrin αvβ6/αvβ8 inhibitors, the current evidence level for AS is conceptual/preclinical only, with no published *in vivo* efficacy data specific to spondyloarthritis (189). As such, these approaches remain firmly within the domain of target discovery and validation.

## 3 Future perspectives and challenges

The integration of spatial multi-omics, encompassing single-cell RNA sequencing (scRNA-seq), spatial transcriptomics, and imaging mass cytometry, represents a groundbreaking approach to elucidating the intricate interplay between macrophage subsets and osteoprogenitor cells in AS. By precisely mapping the spatial distribution and interactions of these cell types within AS lesions across various disease stages, this methodology aims to identify the specific macrophage populations involved in pathological bone formation. Such insights could dramatically enhance our understanding of the etiology of ossification in AS and potentially inform the development of targeted therapeutic strategies.

The substantial heterogeneity in clinical response to biologic and targeted therapies in AS underscores the urgent need for precision medicine approaches. This review propose that macrophage polarization states themselves could serve as a basis for molecular endotyping. For instance, patients with dominant M1-like signatures and elevated ETS2 activity might preferentially respond to JAK inhibitors, whereas those with prominent TGF-β/BMP-driven pro-osteogenic macrophages could benefit from BMP-directed agents or integrin αvβ inhibitors. Large-scale multi-omic profiling (single-cell transcriptomics, proteomics, and epigenomics) of entheseal and synovial macrophages—coupled with machine-learning algorithms—will be essential to define clinically actionable endotypes. Future prospective trials should test whether endotype-matched therapy improves radiographic outcomes compared with current ‘one-size-fits-all’ algorithms.

Moreover, deciphering the dynamics and heterogeneity of macrophage responses throughout the disease progression is critical for personalizing treatment modalities and optimizing intervention timing. The temporal transition from early inflammatory macrophages to those with osteogenic potential underscores the need to capture these shifts in response patterns among patients. To complement this biological framework, advances in biomechanical models, such as organ-on-a-chip systems coupled with in silico simulations, will be instrumental in investigating how mechanical forces influence macrophage behavior and the associated biochemical signaling pathways.

Beyond the TGF−β/BMP axis, other mechanosensitive pathways—most notably the Piezo1 ion channel and the Wnt/β−catenin cascade—have recently emerged as potential drivers of pathological osteogenesis in AS (72). Piezo1 activation by tensile stress promotes autophagic and inflammatory responses in entheseal cells, while dysregulated Wnt signaling may contribute to the uncoupling between inflammation and bone formation. Targeted small−molecule inhibitors against Piezo1 or specific Wnt modulators could theoretically halt spinal fusion independently of inflammatory activity, although such approaches remain largely preclinical. Future studies should explore whether combinatorial targeting of these pathways alongside macrophage−centered strategies yields additive or synergistic benefits. Moreover, the well-established clinical link between inflammatory bowel disease and AS suggests that gut dysbiosis and neoantigen generation may represent upstream triggers of entheseal inflammation. While a full discussion is beyond the scope of this review, we note that future studies should investigate how gut-derived metabolites or microbial products influence macrophage polarization in the enthesis, potentially via modulation of the TGF-β/BMP axis. Characterizing these gut–enthesis interactions could open avenues for microbiome-based diagnostics or dietary interventions. Furthermore, sex-based differences in AS presentation and outcomes remain understudied. Female patients often experience longer diagnostic delays and may exhibit distinct patterns of inflammation and bone formation, possibly reflecting sex-specific modulation of macrophage polarization and TGF-β/BMP signaling. Therefore, the current review advocates that future mechanistic studies—including single-cell analyses of entheseal macrophages—be adequately powered to detect sex-dependent differences. Moreover, clinical trials should enforce gender-balanced enrollment, report outcomes stratified by sex, and validate diagnostic criteria (including MRI scoring and PRO thresholds) specifically for female axSpA phenotypes. Addressing these disparities is essential for equitable and effective precision medicine.

Ultimately, the challenge remains to selectively inhibit pathological ossification while preserving physiological bone regeneration, necessitating both targeted drug delivery systems and the identification of unique pharmacological vulnerabilities inherent to the AS ossification process. By addressing these multifaceted challenges, we can pave the way for more efficacious therapeutic interventions in AS.

## 4 Conclusion

The macrophage-derived TGF-β/BMP signaling axis represents a pivotal molecular intersection in the pathophysiology of AS, mechanistically linking inflammation-driven enthesitis to the pathological endochondral ossification that culminates in spinal fusion. The inherent plasticity of macrophages, coupled with their strategic localization within the mechano-inflammatory entheseal niche, enables these cells to transduce biomechanical stress, hypoxia, and cytokine signals into a sustained osteogenic output. This self-amplifying loop—wherein polarized macrophages both produce and activate TGF-β/BMP ligands—provides a coherent framework for understanding why current anti-cytokine therapies, despite effectively suppressing inflammation, often fail to fully arrest radiographic progression.

Building upon this mechanistic framework, several specific research directions emerge as particularly urgent and actionable. First, spatial multi-omic profiling (integrating single-cell transcriptomics, spatial transcriptomics, and imaging mass cytometry) of macrophage subsets directly within human AS entheseal tissues is critically needed to identify the precise pro-osteogenic subpopulations—beyond current M1/M2 nomenclature—that drive syndesmophyte formation at different disease stages. Second, the development of targeted drug delivery systems, such as nanoparticle- or antibody-conjugated approaches that achieve localized modulation of TGF-β/BMP signaling, should be prioritized to avoid the systemic toxicities (e.g., cardiovascular inflammation, impaired wound healing) associated with broad pathway inhibition. Third, preclinical validation of integrin αvβ6/αvβ8 inhibitors and bispecific agents designed to simultaneously reprogram macrophage polarization and block osteogenic execution represents a promising frontier that requires immediate investigation in relevant animal models of spondyloarthritis. By pursuing these mechanistically grounded research priorities, the field can move beyond passive immunosuppression toward active, localized reprogramming of the disease microenvironment—offering the genuine prospect of halting structural progression in AS.
